# 

**Published:** 1874-08

**Authors:** 


*Vol. XXVIII.* **AUGUST, 1874.** *No. 8.*

THE

### DENTAL REGISTER,

A Monthly Journal Devoted to the Interests of the Profession.

EDITED BY J. TAFT, D. D. S.

AND

PUBLISHED BY SPENCER, CROCKER & CO.,

OHIO DENTAL DEPOT,

168 Race Street, Cincinnati, Ohio.

TERMS: $2.50 Per Annum, in Advance; Single Copies 25c.

